# Danggui Shaoyao San: comprehensive modulation of the microbiota-gut-brain axis for attenuating Alzheimer’s disease-related pathology

**DOI:** 10.3389/fphar.2023.1338804

**Published:** 2024-01-12

**Authors:** Jiawei He, Yijie Jin, Chunxiang He, Ze Li, Wenjing Yu, Jinyong Zhou, Rongsiqing Luo, Qi Chen, Yixiao Wu, Shiwei Wang, Zhenyan Song, Shaowu Cheng

**Affiliations:** ^1^ School of Integrated Chinese and Western Medicine, Hunan University of Chinese Medicine, Changsha, Hunan, China; ^2^ Laboratory of Hunan Province for Integrated Traditional Chinese and Western Medicine on Prevention and Treatment of Cardio-Cerebral Diseases, College of Integrated Traditional Chinese and Western Medicine, Hunan University of Chinese Medicine, Changsha, Hunan, China; ^3^ Office of Science and Technology, Hunan University of Chinese Medicine, Changsha, Hunan, China

**Keywords:** Danggui Shaoyao San, Alzheimer’s disease, 16S rDNA, metabonomics, nicotinate and nicotinamide metabolism, microbial-gut-brain axis

## Abstract

**Background:** Alzheimer’s disease (AD), an age-associated neurodegenerative disorder, currently lacks effective clinical therapeutics. Traditional Chinese Medicine (TCM) holds promising potential in AD treatment, exemplified by Danggui Shaoyao San (DSS), a TCM formulation. The precise therapeutic mechanisms of DSS in AD remain to be fully elucidated. This study aims to uncover the therapeutic efficacy and underlying mechanisms of DSS in AD, employing an integrative approach encompassing gut microbiota and metabolomic analyses.

**Methods:** Thirty Sprague-Dawley (SD) rats were allocated into three groups: Blank Control (Con), AD Model (M), and Danggui Shaoyao San (DSS). AD models were established via bilateral intracerebroventricular injections of streptozotocin (STZ). DSS was orally administered at 24 g·kg^−1^·d^−1^ (weight of raw herbal materials) for 14 days. Cognitive functions were evaluated using the Morris Water Maze (MWM) test. Pathological alterations were assessed through hematoxylin and eosin (HE) staining. Bloodstream metabolites were characterized, gut microbiota profiled through 16S rDNA sequencing, and cortical metabolomics analyzed. Hippocampal proinflammatory cytokines (IL-1β, IL-6, TNF-α) were quantified using RT-qPCR, and oxidative stress markers (SOD, CAT, GSH-PX, MDA) in brain tissues were measured with biochemical assays.

**Results:** DSS identified a total of 1,625 bloodstream metabolites, predominantly Benzene derivatives, Carboxylic acids, and Fatty Acyls. DSS significantly improved learning and spatial memory in AD rats and ameliorated cerebral tissue pathology. The formulation enriched the probiotic Ligilactobacillus, modulating metabolites like Ophthalmic acid (OA), Phosphocreatine (PCr), Azacridone A, Inosine, and NAD. DSS regulated Purine and Nicotinate-nicotinamide metabolism, restoring balance in the Candidatus Saccharibacteria-OA interplay and stabilizing gut microbiota-metabolite homeostasis. Additionally, DSS reduced hippocampal IL-1β, IL-6, TNF-α expression, attenuating the inflammatory state. It elevated antioxidative enzymes (SOD, CAT, GSH-PX) while reducing MDA levels, indicating diminished oxidative stress in AD rat brains.

**Conclusion:** DSS addresses AD pathology through multifaceted mechanisms, encompassing gut microbiome regulation, specific metabolite modulation, and the mitigation of inflammation and oxidative stress within the brain. This holistic intervention through the Microbial-Gut-Brain Axis (MGBA) underscores DSS’s potential as an integrative therapeutic agent in combatting AD.

## 1 Introduction

Alzheimer’s disease (AD), an intricate neurodegenerative condition, is clinically characterized by a progressive decline in memory and cognitive functionality, stemming from a spectrum of factors. The global prevalence of dementia, estimated at approximately 50 million individuals in 2018 by Alzheimer’s Disease International, is projected to double by 2050, with a significant burden expected in low- and middle-income countries (Alzheimers Disease International, 2018). Despite the alarming trends, the comprehensive etiology of AD remains elusive, with hallmark pathological features including amyloid-β (Aβ) plaques, neurofibrillary tangles (NFTs) comprised of hyperphosphorylated Tau protein, neuronal loss, and persistent brain inflammation (Sche et al., 2021). Accordingly, research focusing on the pathogenesis, preventive strategies, and therapeutic interventions for AD is critically essential.

The multifaceted nature of Traditional Chinese Medicine (TCM) and botanical drugs, due to their multiple components and targets, is gaining global recognition. TCM adopts a holistic approach, aimed at restoring the body’s overall functional equilibrium. Such interventions have shown promising results in rectifying metabolic imbalances, a common phenomenon in AD. By addressing both the symptoms and underlying imbalances, TCM offers a nuanced, integrative approach to AD treatment (Tang et al., 2008). Danggui Shaoyao San (DSS), a TCM formulation attributed to Zhang Zhongjing and comprising *Angelica sinensis* (*Oliv.*) *Diels* (*Danggui*), *Paeonia lactiflora Pall* (*Shaoyao*), *Atractylodes macrocephala Koidz* (*Baizhu*), *Poria cocos* (*Schw*.) *Wolf* (*Fuling*), *Alisma orientalis* (*Sam*.) *Juzep* (*Zexie*), and *Ligusticum chuanxiong Hort* (*Chuanxiong*), stands as a promising candidate to mitigate AD progression (Kim and Cho, 2020; Fu et al., 2015). Studies indicate DSS’s neuroprotective mechanisms, notably enhancing antioxidant enzyme activity and mitigating type IV collagen accumulation, thus exhibiting antioxidative potential (Liu et al., 2012; Huang et al., 2012). DSS has been shown to regulate neurotransmitter levels and enhance SOD activity across various brain regions, implicating its role in modulating brain tissue metabolism (Ueda et al., 1996). Furthermore, DSS exerts anti-inflammatory effects, suppressing nitric oxide (NO) levels and proinflammatory cytokines in the hippocampus and attenuating neuronal apoptosis (Song et al., 2021). It modulates apoptosis-related protein expression, fostering neuronal survival (Lan et al., 2012), and improves cognitive functions, mitigating Aβ-induced damage (Lu, 2001).

The distinct attributes of DSS, including its holistic therapeutic advantages, neuroprotective efficacy, neurotransmitter balancing, anti-inflammatory potential, biochemical marker normalization, comprehensive neurotoxicity protection, and cognitive function enhancement, are pivotal (Ueda et al., 1996; Lu, 2001; Huang et al., 2012; Lan et al., 2012; Liu et al., 2012; Fu et al., 2015; Song et al., 2021). Prior research has extensively explored DSS’s anti-AD effects using techniques like ultrahigh-performance liquid chromatography/high-resolution mass spectrometry (UPLC-HRMS) (Zhang et al., 2021), enabling the accurate identification of natural products. This technique enables the separation of multiple substances in a high-throughput manner. High-resolution mass spectrometry analysis allows for the accurate deduplication of known natural products using commercial databases such as the Dictionary of Natural Products (DNP), LipidMaps, and KEGG (Chagas-Paula et al., 2015; Cao et al., 2021). Our previous studies have provided a detailed description of the extraction and identification of DSS (Song et al., 2020).

Moreover, the 16S rDNA sequencing technique has provided insights into the gut microbiota’s role in AD, revealing significant differences in microbial composition between AD patients and healthy elderly individuals (Wang et al., 2022; Li et al., 2019; Liu et al., 2019). Some plant-derived small molecules, such as icariin, can alter the gut microbiota composition in APP/PS1 mice, leading to changes in metabolites and reflecting their anti-AD mechanisms (Liu et al., 2023). The impact of DSS on the gut microbiota and its potential implications for AD are of paramount significance, particularly concerning gram-negative bacteria and the subsequent release of bacterial lipopolysaccharides (LPS). This intricate relationship has been elucidated in recent literature (Sharma and Martins, 2023), pointing to the pivotal role of DSS in influencing the composition of gut microbiota and, consequently, modulating the release of LPS.

Metabolomics, a rapidly advancing field, offers insights into disease-induced metabolic disturbances (Huo et al., 2020). Untargeted mass spectrometry enables comprehensive and systematic exploration of metabolites from biological organisms, providing a reliable hypothesis-generating method to identify and validate candidate metabolomic biomarkers (Su et al., 2023). Utilizing untargeted mass spectrometry, our study aims to provide a comprehensive analysis of pathophysiological mechanisms in AD (Reveglia et al., 2021). Early screening and diagnosis using metabolomic biomarkers are promising, as changes in brain tissue metabolites often precede clinical symptom manifestation (Lista et al., 2023).

This study establishes an AD rat model treated with DSS, aiming to unravel the complexity of AD biology and DSS’s regulatory network in therapy. Through these investigations, we aspire to contribute novel insights into the anti-AD effects of DSS, highlighting its comprehensive impact on cognitive function, histopathology, gut microbiota, and metabolomic profiles.

## 2 Materials

### 2.1 Drugs and reagents

The DSS was composed of *A. sinensis* (*Oliv.*) Diels (Danggui) 120 g, *Paeonia lactiflora* Pall (Shaoyao) 640 g, *Atractylodes macrocephala* Koidz (Baizhu) 160 g, *Poria cocos (Schw.)* Wolf (Fuling) 160 g, *Alisma orientalis (Sam.)* Juzep (Zexie) 320 g, and *Ligusticum chuanxiong* Hort (Chuanxiong) 320 g. All botanical drugs materials were procured from the Traditional Chinese Medicine Pharmacy at the First Affiliated Hospital of Hunan University of Chinese Medicine. Streptozotocin (STZ) was obtained from Solarbio (Catalog No. S8050). The following reagents were used: water (Fisher Chemical, CAS No. 1336-21-6, LC-MS grade), acetonitrile (CNW Technologies, CAS No. 75-05-8, LC-MS grade), methanol (CNW Technologies, CAS No. 67-56-1, LC-MS grade), formic acid (Honeywell, LC-MS grade), ammonium acetate (SIGMA-ALDRICH, CAS No. 631-61-8, LC-MS grade). For PCR product purification, AMPure XT beads were obtained from Beckman Coulter Genomics (Danvers, MA, United States). PCR product quantification was performed using the Qubit quantification assay (Invitrogen, United States). Assay kits for MDA,CAT,SOD,GSH-PX [Nanjing Jiancheng Bioengineering Institute, China (Catalog No. A003-1, A007-1-1, A001-3, A005-1)].

### 2.2 Animals

Thirty male Sprague-Dawley rats (150–200 g), obtained from Hunan SJA Laboratory Animal Co., Ltd. (SCXK (Xiang) 2021-0002), were acclimatized and housed under controlled conditions at Hunan University of Chinese Medicine’s Animal Experimental Center. Ethical approval (No. LLBH-202103180001) from the university’s Ethics Committee underlined the commitment to humane animal treatment. Rats were randomly divided into control, model, and DSS groups, receiving standardized care throughout the study.

### 2.3 Instruments

The SMART 3.0 Small Animal Behavioral Recording and Analysis System (Ruiwo Life Science & Technology Co., Ltd.); Paraffin microtome (Thermo Fisher, United States); Al+ confocal laser scanning microscope (Nikon, Japan); Low-temperature high-speed centrifuge (Eppendorf Centrifuge 5430 R); Centrifuge (Thermo Fisher Scientific, Heraeus Fresco 17); Mass spectrometer (Thermo Fisher Scientific, Q-Exactive HFX); Ultra-high-performance liquid chromatography (Thermo Fisher Scientific, Vanquish); Chromatographic column (ACQUITY UPLC HSS T3, 2.1 mm × 100 mm, 1.8 µm); Balance (Sartorius, BSA124S-CW); Ultrasonic processor (Shenzhen Redbond Electronics Co., Ltd., PS-60AL); Agilent 2100 Bioanalyzer (Agilent, United States); Illumina (Kapa Biosciences, Woburn, MA, United States); Paraffin microtome (Thermo Fisher, United States); Al+ confocal laser scanning microscope (Nikon, Japan); SorvaliTM LegendTM Micro 17R microcentrifuge (Thermo Fisher, United States); Cytation3 Multifunctional Microplate Reader (Bio-Tek, United States); T100™ Thermal Cycler real-time quantitative polymerase chain reaction amplification instrument, among others.

## 3 Methods

### 3.1 Drug preparation

The amalgamation of the six metabolites botanical drugs of DSS commenced by subjecting them to a meticulous process. The botanical drugs ensemble was intricately mixed and subjected to eight cycles of soaking in distilled water, employing a volumetric to weight ratio (v/w), over a duration of 1.5 h. Subsequently, the concoction underwent a precisely orchestrated sequence: a 0.5-h boiling phase followed by an additional hour of simmering. Post this intricate process, the resultant filtrate was meticulously collected, while the residual botanical drugs matter underwent a subsequent extraction iteration. This iterative extraction, consistent with prior procedural delineations, deviated only in the rinsing frequency - limited to six cycles (v/w) in distilled water. The aqueous elixir derived from DSS underwent a concentration procedure utilizing a rotary evaporator, culminating in an ultimate concentration of 1 g/mL, mirroring the dry weight of the original botanical drugs metabolites (Song et al., 2020; Luo et al., 2023).

### 3.2 Animal modeling

STZ was dissolved in citrate buffer to prepare STZ injection solution at a dose of 2.4 mg/kg. Modeling commenced after a 12-h fast. Anesthesia was induced with 10% chloral hydrate, and using a stereotaxic apparatus and a microinjector, the lateral ventricles of the rat brain were located with reference to the “Rat Brain Stereotaxic Atlas.” STZ was injected at a uniform rate of 5 μL over 5 min into each lateral ventricle. The control group received an equivalent volume of saline. After injection, the needle was retained to prevent STZ leakage. Following needle withdrawal, the incision was sutured, and the rats were returned to their cages after recovery. The success of modeling was evaluated using the MWM test (Song et al., 2021).

### 3.3 Animal grouping and drug intervention

Thirty rats were randomly divided into three groups: Con,M, and DSS. Intervention began 3 days after modeling, and the treatment lasted for 14 days. The model group received an equivalent volume of saline by gavage. The dosage for the DSS group was determined based on the overall botanical drugs weight of DSS at 24 g·kg^−1^·d^−1^ (weight of raw herbal materials), adjusted for body surface area and previous preliminary studies (Yang et al., 2023).

### 3.4 Morris water maze experiment

MWM test was employed to assess spatial learning and memory. Using the SMART 3.0 Small Animal Behavioral Recording and Analysis System, the pool was divided into four quadrants, each marked with distinct identifiers. The target quadrant was designated as the first quadrant, and the escape platform was fixed 1 cm below the water surface in this quadrant. The MWM test included the place navigation experiment and the spatial probe test. In the place navigation experiment, rats were placed into the water from various starting points along the pool wall, and the time taken for a rat to climb onto the platform and stay for 2 s was recorded. If a rat failed to reach the platform within 60 s, it was guided to the platform for a 10-s stay to assist memory, with two learning sessions per day. In the spatial probe test on the 6th day, the escape platform was removed, and as previously described, the time spent in the first quadrant and the number of times rats crossed the platform within 60 s were recorded.

### 3.5 Sample collection

Rats were anesthetized with 3% pentobarbital sodium based on their body weight. After fixing the rat limbs, a U-shaped incision was made in the abdominal cavity. Blood was collected from the abdominal aorta using a blood collection needle, approximately 3 mL per rat. Pre-cooled 50 mL physiological saline was injected into the left ventricle at 4°C, and the right atrium was opened. Successful heart perfusion was indicated by visceral whitening. Rat brain tissues were harvested, and the left and right hemispheres were separated. The left hemisphere was fixed in paraformaldehyde, and the right hemisphere was dissected into cortex and hippocampus, then stored in liquid nitrogen before transferring to a −80°C freezer. At least five fecal pellets were collected from each rat’s rectum, placed in frozen tubes, and stored in a −80°C freezer until further analysis. Blood was centrifuged at 3,000 rpm for 15 min, and the upper layer serum was stored at −80°C.

### 3.6 HE staining

After fixation, the left hemisphere was embedded in paraffin, cut into 3 μm thick slices using a paraffin microtome. The sections were dewaxed and rehydrated, stained with hematoxylin and eosin (HE) for 5 min, differentiated in 0.5% hydrochloric acid ethanol for 1 min, counterstained with eosin for 1 min, dehydrated, air-dried, and coverslipped with neutral gum at room temperature.

### 3.7 RT-qPCR analysis

RNA from the left hippocampal tissue was extracted using the TRIzol method. The RNA concentration was measured, and the obtained RNA was stored at −80°C. cDNA synthesis was performed using the NovoScript^®^ mRNA First-Strand cDNA Synthesis kit. The first-strand cDNA synthesis reaction was conducted at the specified temperature and time using 1,000 ng of RNA in a 20 μL reaction system, and the synthesized cDNA was stored at −20°C. For the PCR stage, Primer Premier 6.0 was utilized to design primers with a Tm of 55°C ± 5°C and an amplicon length of 100-300 bp (Table 1). SYBR Green dye was used for qPCR to detect the mRNA expression levels of IL-1β, IL-6, and TNF-α, with β-actin as the internal reference. The 2^−ΔΔcq^ method was employed to calculate the relative expression levels of each mRNA.

**TABLE 1:** Primer sequence.

Primer	Forward primer (5´→3′)	Reverse primer (3´→5′)	Length/bp
IL-1β	CTC​ATT​GTG​GCT​GTG​GAG​AAG	ACA​CTA​GCA​GGT​CGT​CAT​CAT	148
IL-6	CCA​GCC​AGT​TGC​CTT​CTT​G	AAT​TAA​GCC​TCC​GAC​TTG​TGA​A	139
TNF-α	AGA​TGT​GGA​ACT​GGC​AGA​GG	CAC​GAG​CAG​GAA​TGA​GAA​GAG	100

### 3.8 Detection of oxidative stress biomarkers

Samples were subjected to conventional homogenization (homogenization medium: PBS, 0.01 M, pH 7.4). After homogenization, the samples were centrifuged at 4°C, 10,000 × g for 10 min, and the supernatant was collected and kept on ice for further analysis. A portion of the supernatant was reserved for protein concentration determination using the BCA method. The levels of MDA, CAT, SOD, and GSH-PX were measured using assay kits from Jiangsu Jiancheng Bioengineering Institute (A003-1, A007-1-1, A001-3, A005-1) following the manufacturer’s instructions. The measurements were performed based on the protein concentration determined by the BCA method.

### 3.9 Identification of circulating components in DSS

The UPLC-HRMS method was employed to detect the chemical composition in DSS (DSS_Pre), blank serum (CON), and post-administration serum (DSS). Secondary mass spectrometry spectra were compared with a mass spectrometry database to identify the components with higher content in DSS. Subsequently, the circulating chemical components in post-administration serum were analyzed.

#### 3.9.1 Preparation of test solution

Approximately 0.02 g of Danggui Shaoyao San (DSS_Pre) powder sample was placed in a centrifuge tube (2 mL), followed by the addition of 1 mL of 70% methanol-water solution. The mixture was vortexed, sonicated for 30 min, centrifuged at 16,000 g at 4°C for 15 min, and the supernatant was collected. The supernatant was then vacuum freeze-dried, and the residue was reconstituted with 0.6 mL of 40% methanol-water solution. After vortex mixing and centrifugation (16,000 g at 4°C for 15 min), the supernatant was collected.

#### 3.9.2 Preparation of serum samples

A 200 μL serum sample was mixed with 800 μL methanol, vortexed for 60 s, allowed to stand at −20°C for 30 min, and then centrifuged at 16,000 g at 4°C for 20 min. The supernatant was collected, vacuum-dried, and the residue was reconstituted with 100 μL of 40% methanol-water solution. After vortexing and centrifugation (16,000 g at 4°C for 15 min), the supernatant was collected.

#### 3.9.3 Preparation of blank serum + DSS_Pre samples

A 200 μL blank serum was mixed with 50 μL of DSS_Pre test solution. The mixture was then combined with 800 μL methanol, vortexed for 60 s, allowed to stand at −20°C for 30 min, and centrifuged at 16,000 g at 4°C for 20 min. The supernatant was collected, vacuum-dried, and the residue was reconstituted with 100 μL of 40% methanol-water solution. After vortexing and centrifugation (16,000 g at 4°C for 15 min), the supernatant was collected.

#### 3.9.4 Analytical procedure

Precisely, 4 μL of each blank serum sample, drug-administered serum sample, and blank serum + DSS_Pre sample, as well as 2 μL of the DSS_Pre test solution, were aspirated for LC-MS injection. Each batch of blank serum and drug-administered serum samples was injected once, and the blank serum + DSS_Pre sample was injected three times, while the DSS_Pre sample was injected five times.

#### 3.9.5 Compound identification

The raw data files in .raw format were converted to. mzXML format using the ProteoWizard. XCMS software was employed for peak alignment, retention time correction, and peak extraction. The data extracted by XCMS were then matched with standard spectrum databases for structural identification. The matching of secondary mass spectra (MS2) was primarily reflected in the MS2 fragment similarity score. The higher the score (with a total score of 1), the more reliable the identification results (Dunn et al., 2013; Liang et al., 2020). A score of 0.7 or higher is generally considered reliable. Therefore, the parameters were set as MS1 difference less than 15 ppm and MS2 fragment similarity score greater than 0.7 (Said et al., 2020; Fan et al., 2020).

### 3.10 16S rRNA gene sequencing analysis

#### 3.10.1 Extraction of intestinal content DNA and PCR amplification

Primers with specific barcodes for each sample and sequencing universal primers were tagged at the 5′ ends (Table 2). PCR amplification was carried out in a total volume of 25 μL reaction mixture containing 25 ng of template DNA, 12.5 μL of PCR Premix, 2.5 μL of each primer, and PCR-grade water to adjust the volume. The PCR conditions for amplifying prokaryotic 16S fragments included an initial denaturation at 98°C for 30 s; 32 cycles of denaturation at 98°C for 10 s, annealing at 54°C for 30 s, and extension at 72°C for 45 s; followed by a final extension at 72°C for 10 min. The PCR products were confirmed by 2% agarose gel electrophoresis. Throughout the DNA extraction process, ultrapure water was used as a negative control instead of a sample solution to eliminate the possibility of false-positive PCR results.

**TABLE 2:** PCR amplification and 16S rDNA sequencing.

Region	Primers
V3-V4 Logue et al., (2016)	341F (5′-CCTACGGGNGGCWGCAG-3′)
	805R (5′-GACTACHVGGGTATCTAATCC-3′)
Archae Takai and Horikoshi, (2000)	F (5′-GYGCASCAGKCGMGAAW-3′)
	R (5′-GGACTACHVGGGTWTCTAAT-3′)
V4 (Walters et al., (2015)	515F (5′-GTGYCAGCMGCCGCGGTAA-3′)
	806R (5′- GGACTACHVGGGTWTCTAAT-3′)
V4-V5	F (5′-GTGCCAGCMGCCGCGG-3′)
	R (5′-CCGTCAATTCMTTTRAGTTT-3′)

The PCR products were purified using AMPure XT beads (Beckman Coulter Genomics, Danvers, MA, United States) and quantified with Qubit (Invitrogen, United States). Amplicon pools were prepared for sequencing, and the size and quantity of the amplicon library were assessed on an Agilent 2100 Bioanalyzer (Agilent, United States) and with the Library Quantification Kit for Illumina (Kapa Biosciences, Woburn, MA, United States), respectively. The libraries were sequenced on the NovaSeq PE250 platform.

#### 3.10.2 Data analysis

Samples were sequenced on an Illumina NovaSeq platform following the manufacturer’s recommendations. Paired-end reads were assigned to samples based on their unique barcode and truncated by removing the barcode and primer sequence. The paired-end reads were merged using FLASH. Quality filtering on the raw reads was performed under specific conditions to obtain high-quality clean tags according to fqtrim (v0.94). Chimeric sequences were filtered using Vsearch software (v2.3.4). After dereplication using DADA2 (Callahan et al., 2016), we obtained feature tables and feature sequences. Alpha diversity and beta diversity were calculated by normalizing to the same sequences randomly. Then, according to the SILVA (release 138) classifier, feature abundance was normalized using the relative abundance of each sample. Alpha diversity was analyzed using five indices, including Chao1, Observed species, Goods coverage, Shannon, and Simpson, with QIIME2. Beta diversity was calculated using QIIME2 (Lozupone et al., 2011), and the graphs were generated using the R package. Blast was used for sequence alignment, and the feature sequences were annotated with the SILVA database for each representative sequence. Differential analysis using Linear Discriminant Analysis Effect Size (LEfSe) was employed to identify significantly different taxa (Segata et al., 2011). The prediction of Clusters of Orthologous Groups (COG) functional profiles was conducted using PICRUSt software. Other diagrams were generated using the R package (v3.5.2).

### 3.11 LC–MS

#### 3.11.1 Metabolite extraction

A total of 25 mg of sample was weighed into an EP tube, and 500 μL of the extraction solution (methanol:acetonitrile:water = 2:2:1, with an isotopically-labelled internal standard mixture) was added. The samples were then homogenized at 35 Hz for 4 min and sonicated for 5 min in an ice-water bath. This homogenization and sonication cycle was repeated three times. Subsequently, the samples were incubated for 1 h at −40°C and centrifuged at 12,000 rpm [RCF = 13,800(×g), R = 8.6 cm] for 15 min at 4°C. The resulting supernatant was transferred to a fresh glass vial for analysis. A quality control (QC) sample was prepared by mixing an equal aliquot of the supernatants from all the samples.

#### 3.11.2 LC-MS/MS analysis

LC-MS/MS analyses were conducted using a UHPLC system (Vanquish, Thermo Fisher Scientific) with a UPLC BEH Amide column (2.1 mm × 100 mm, 1.7 μm) coupled to the Q Exactive HFX mass spectrometer (Orbitrap MS, Thermo) (Wang et al., 2016). The mobile phase comprised 25 mmol/L ammonium acetate and 25 ammonia hydroxide in water (pH = 9.75) (A) and acetonitrile (B). The auto-sampler temperature was set at 4°C, and the injection volume was 2 μL.

The QE HFX mass spectrometer was chosen for its capability to acquire MS/MS spectra on information-dependent acquisition (IDA) mode, controlled by the acquisition software (Xcalibur, Thermo). In this mode, the acquisition software continuously evaluates the full scan MS spectrum. The ESI source conditions were configured as follows: sheath gas flow rate at 30 Arb, Aux gas flow rate at 25 Arb, capillary temperature at 350°C, full MS resolution at 120,000, MS/MS resolution at 7,500, collision energy at 10/30/60 in NCE mode, and spray voltage at 3.6 kV (positive) or −3.2 kV (negative), respectively.

#### 3.11.3 Data preprocessing and annotation

The raw data were converted to the mzXML format using ProteoWizard and processed with an in-house program developed using R and based on XCMS for peak detection, extraction, alignment, and integration (Smith et al., 2006). Subsequently, an in-house MS2 database (BiotreeDB) was employed for metabolite annotation. The annotation cutoff was set at 0.3.

### 3.12 Analysis of gut microbiota-metabolite correlation

We employed correlation analysis to identify different metabolites involved in key metabolic pathways in the CON group, M group, and DSS group. The top 45 most abundant species in the gut microbiota were determined across these three groups. Subsequently, Spearman correlation analysis and a heatmap were utilized to assess the correlation between gut microbiota composition and metabolite levels.

### 3.13 Statistical analysis

Statistical analysis was performed using SPSS 25.0 software. Quantitative data are presented as mean ± SEM and analyzed using one-way analysis of variance (ANOVA). Graphs were generated using Graphpad 8.0.1. Differences were considered statistically significant when the *p*-value was less than 0.05.

## 4 Result

### 4.1 Analysis of DSS components

This study establishes an AD rat model treated with DSS, aiming to unravel the complexity of AD biology and DSS's regulatory network in therapy. Through these investigations, we aspire to contribute novel insights into the anti-AD effects of DSS, highlighting its comprehensive impact on cognitive function, histopathology, gut microbiota, and metabolomic profiles.

**FIGURE 1:**
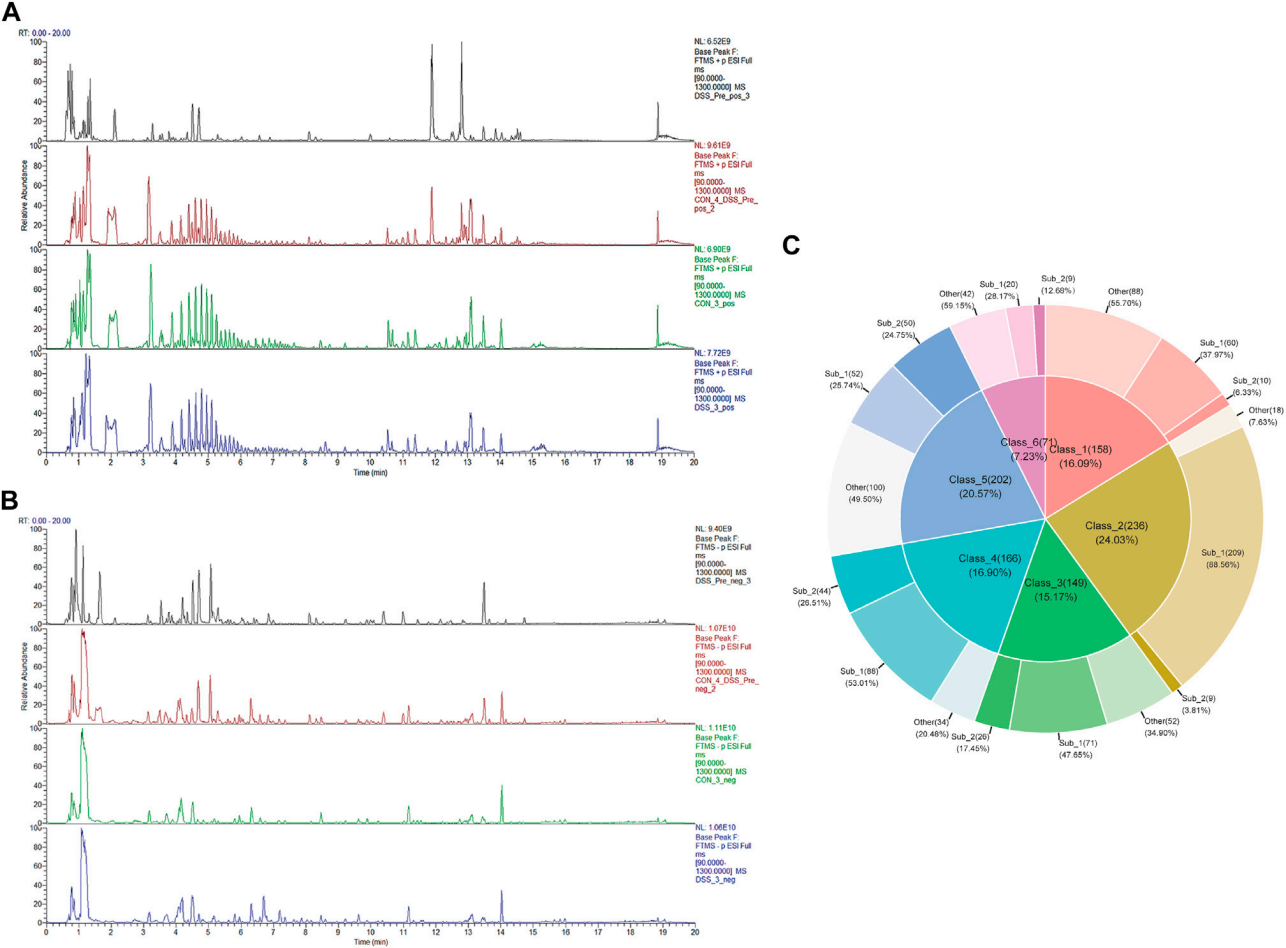
Identification of DSS metabolites in Bloodstream. **(A)** BPC of various sample groups under positive ion mode, from top to bottom: DSS (DSS_Pre), blank serum with DSS_Pre (CON4+ DSS_Pre), blank serum (CON), and administered serum (DSS). **(B)** BPC of various sample groups under negative ion mode, from top to bottom: DSS (DSS_Pre), blank serum with DSS_Pre (CON4+ DSS_Pre), blank serum (CON), and administered serum (DSS). **(C)** Top 6 Metabolite categories and their proportions of subclasses.

To delineate the active chemical constituents of Danggui Shaoyao San (DSS), an advanced UPLC-HRMS technique was harnessed. This approach enabled the comprehensive profiling of components in various sample matrices: blank serum (CON), DSS-administered serum (DSS), and DSS itself (DSS_Pre). Comparative analysis of base peak chromatograms (BPC) in both positive and negative ion modes, as illustrated in Figures 1A, B, facilitated the identification of prominent chromatographic peaks. These peaks, meticulously inspected for shape and secondary spectral characteristics, were systematically cataloged. The identification process involved a rigorous comparison with established spectral databases pertinent to TCM. This analytical strategy culminated in the identification of an extensive array of 1,625 chemical components within DSS and its post-administration serum. Following the classification criteria delineated in the ClassyFire literature (Feunang and Yannick, 2016), these components were classified into 120 distinct categories, detailed in Supplementary File S1. Furthermore, Figure 1C elucidates the six major compound categories, delineating their subclasses and proportional distribution, thereby providing a comprehensive chemical landscape of DSS.

### 4.2 Improvement of learning and memory abilities, as well as pathological changes in brain tissues, by DSS in AD rats

**FIGURE 2:**
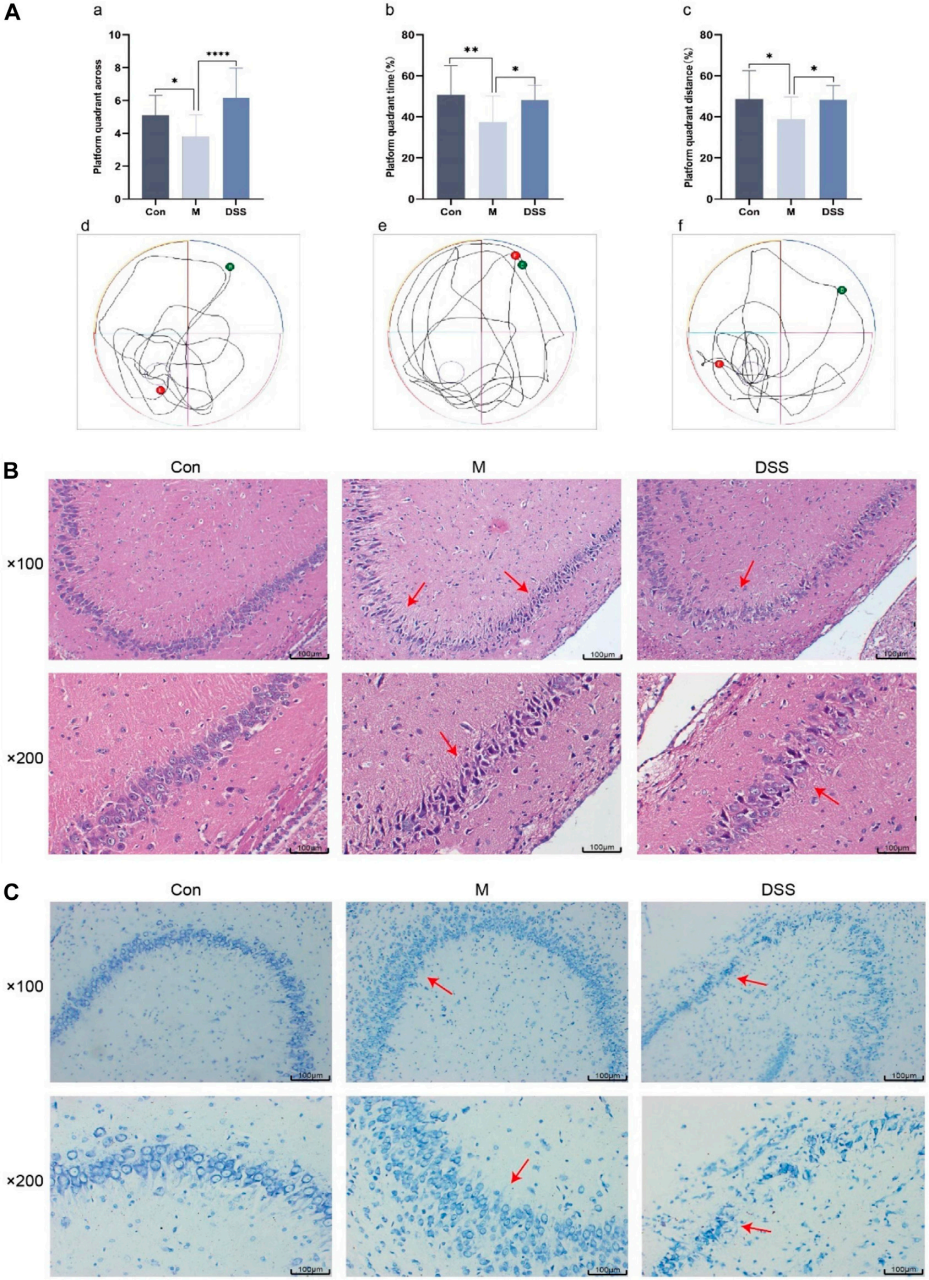
Behavioral, Brain Tissue HE and Nissl Staining in Rats. **(A)** Morris Water Maze Experiment. (a) Number of platform crossings in the spatial exploration test. (b) Time spent in the platform quadrant during the spatial exploration test. (c) Distance traveled in the platform quadrant during the spatial exploration test. (d) Movement trajectory of Con group rats. (e) Movement trajectory of M group rats. (f) Movement trajectory of DSS group rats. **(B)** Rat brain tissue HE staining. **(C)** Rat brain tissue Nissl staining. *p < 0.05; **p < 0.01; ***p < 0.001; ****p < 0.0001.

The efficacy of DSS in AD rats was evaluated through a spatial exploration experiment following a 5-day spatial navigation trial. In this assessment (Figure 2A), the AD rats, particularly in the model group, displayed diminished platform crossings and reduced time and distance in the platform quadrant, indicative of impaired spatial memory. Contrastingly, the DSS group showed enhanced performance with increased platform crossings and extended time and distance in the quadrant, suggesting improved spatial memory post DSS treatment.

Histopathological changes were examined using HE staining, focusing on the hippocampus, a region vulnerable to early AD damage (Mu and H Gage, 2011). The HE staining results (Figure 2B) revealed orderly, spherical neural cells with intact membranes in the control group. In contrast, the model group exhibited disorganized, variably sized neural cells, with a noticeable reduction in number. DSS treatment appeared to restore neural integrity, reversing signs of cell necrosis.

Nissl staining (Figure 2C) further corroborated these observations. In the control group, neurons appeared normal with regular, densely stained Nissl bodies. The model group showed neuronal atrophy, decreased Nissl bodies, and disrupted nuclear integrity. Post-DSS intervention, a marked improvement in neuronal organization, Nissl body count, and cellular morphology was observed, indicating DSS’s neuroprotective effect.

### 4.3 DSS modulates the composition of gut microbiota in AD rats

**FIGURE 3:**
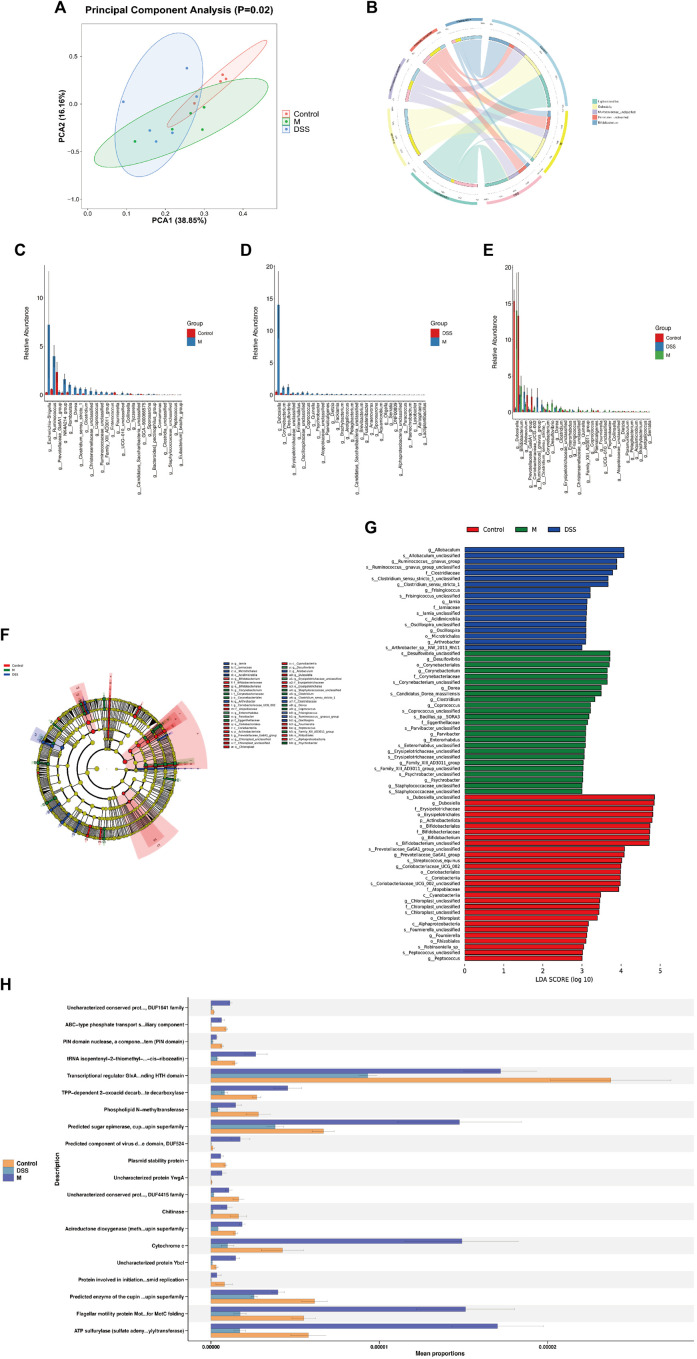
(Continued).

To investigate the potential correlation between the anti-AD effects of DSS and alterations in the gut microbiota, 16S rRNA gene sequencing was conducted on fecal samples from the Con, M, and DSS3 groups. Alpha diversity indices, including Chao1, observed species, Shannon, and Simpson, were employed to assess species richness and diversity among the groups (Supplementary Figures S1A–D). The results indicated a reduction in the number of microbial species in the gut of AD rats after DSS treatment, as evident from Chao1 and observed species indices. Shannon and Simpson indices illustrated an improvement in microbial diversity with DSS intervention, with a decrease in diversity in the DSS group approaching that of the Con group, compared to the M group. Beta diversity analysis, as shown by PCA (Figure 3A) and PCoA (Supplementary Figures S1E, F), revealed significant differences in microbial species composition among the three groups.

To further explore the general composition of the gut microbiota in the three groups, we selected the top 30 abundant species, classified them by genus, and examined the similarity in classification among bacteria. Results indicated that Ligilactobacillus, Dubosiella, and Bifidobacterium were predominant genera in the Con group, Dubosiella, Clostridia UCG-014 unclassified, and Firmicutes unclassified in the M group, and Ligilactobacillus, Akkermansia, and Muribaculaceae unclassified in the DSS group (Supplementary Figure S1G). Analysis of the distribution of the top five abundant taxa (Figure 3B; Table 3) revealed the dominance of Ligilactobacillus in the Control group, a significant decrease in the M group, and a substantial recovery in abundance after DSS intervention. Dubosiella was highly abundant in the M group but significantly reduced in the DSS group. In the analysis of significant differences (Figures 3C, D; Supplementary File S2), six taxa in the M group showed decreased abundance compared to the Control group, while 20 taxa exhibited increased abundance. Notably, Prevotellaceae Ga6A1 group showed a decreasing trend, and Escherichia-Shigella displayed an increasing trend at the abundance level. When comparing the DSS and M groups, all 46 taxa in the DSS group exhibited reduced abundance. Notably, Dubosiella showed the most significant reduction in abundance. In the comparison of all three groups (Figure 3E; Supplementary File S3), Dubosiella abundance decreased in the M group and continued to show a decreasing trend after DSS intervention.

**TABLE 3:** Distribution proportions of top 5 dominant genera at the genus level.

Genus	Control	M	DSS
Ligilactobacillus	20.75	2.11	15.05
Dubosiella	15.36	14.02	0.51
Muribaculaceae_unclassified	8.44	5.70	7.43
Firmicutes_unclassified	3.48	7.84	6.09
Bifidobacterium	13.30	3.57	2.41

Through Kruskal–Wallis rank-sum tests and Wilcoxon rank-sum tests, we identified significantly different species and assessed their contribution using Linear Discriminant Analysis (LDA) (Figures 3F, G; Supplementary File S4). The most differentially abundant species varied among the groups; Desulfovibrio contributed the most in the M group, Allobaculum in the DSS group, and Dubosiella in the Control group. Functional predictions using PICRUSt2 revealed changes in COG functional profiles (Figure 3H; Supplementary File S5). Compared to the Con group, the M group showed a decrease in functions related to ABC-type phosphate transport system, auxiliary component, PIN domain nuclease, a component of toxin-antitoxin system (PIN domain), Transcriptional regulator GlxA family. Conversely, functions related to Uncharacterized conserved protein YjgD, DUF1641 family, tRNA isopentenyl-2-thiomethyl-A-37 hydroxylase MiaE (synthesis of 2-methylthio-cis-ribozeatin), TPP-dependent 2-oxoacid decarboxylase, including indolepyruvate decarboxylase, increased. When comparing the DSS and M groups, all top 20 functions showed a decrease in the DSS group, particularly involving various metabolic processes such as glycolipid metabolism, redox processes, gene regulation, bacterial motility, antiviral responses, and post-translational modification. These results suggest that DSS can mediate its anti-AD effects by modulating the abundance and functionality of the gut microbiota in AD rats.

### 4.4 DSS reverses metabolic disruptions in the brains of AD rats

**FIGURE 4:**
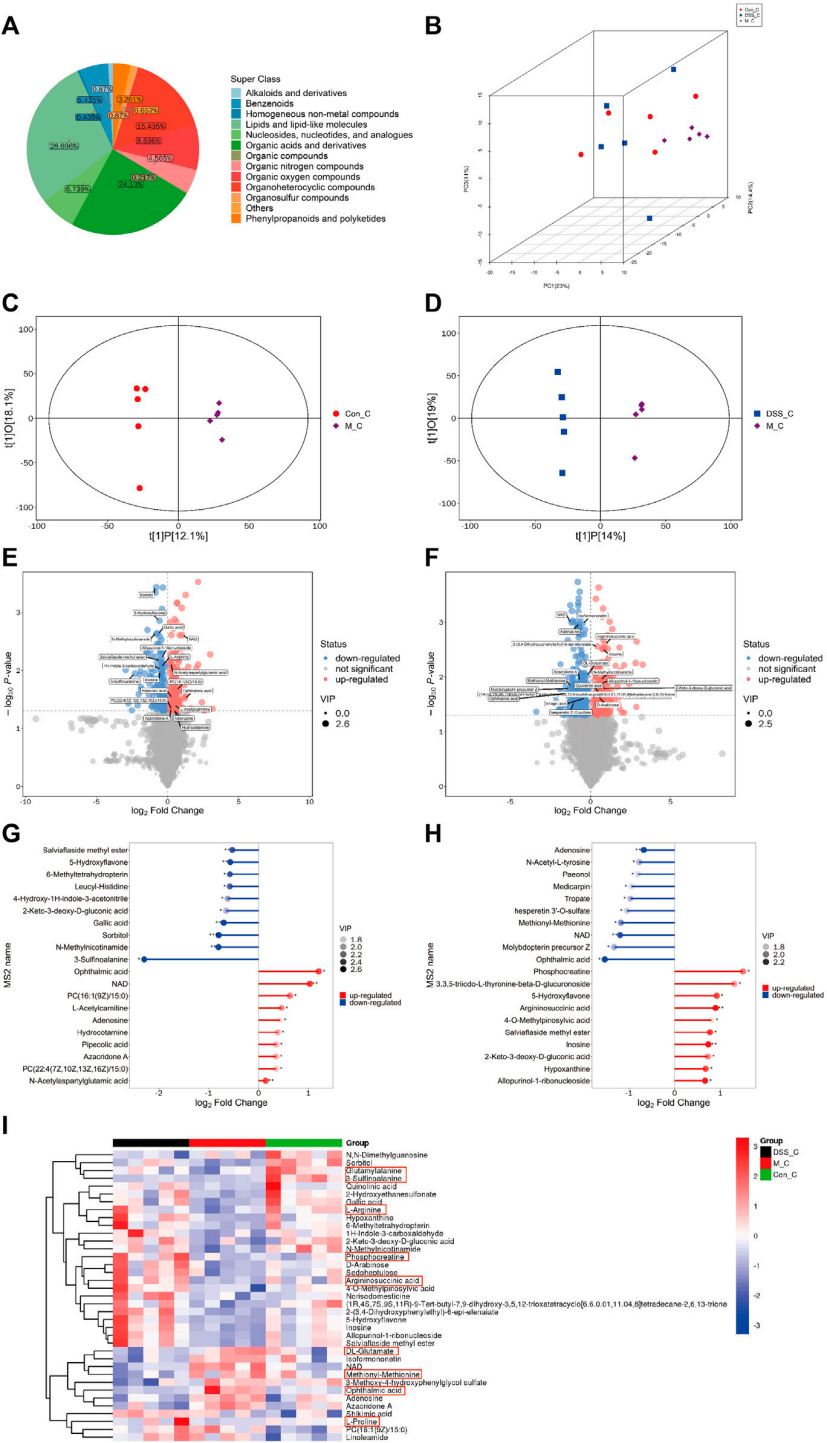
Metabolomic Analysis of Rat Brain Tissues **(A)** Pie chart depicting metabolite classification and distribution. **(B)** PCA analysis of the three groups. **(C)** OPLS-DA analysis between the Con and M groups. **(D)** OPLS-DA analysis between the M and DSS groups. **(E)** Volcano plot illustrating metabolic differences between the Con and M groups. **(F)** Volcano plot illustrating metabolic differences between the M and DSS groups. **(G)** Metabolic pathway analysis based on differential metabolites between the Con and M groups. **(H)** Metabolic pathway analysis based on differential metabolites between the M and DSS groups. **(I)** Hierarchical cluster analysis heatmap showing intergroup differences. *p < 0.05; **p < 0.01; ***p < 0.001; ****p < 0.0001.

Our findings indicate that DSS influences the metabolic characteristics of the gut microbiota in AD rats. We further conducted untargeted metabolomics on brain tissues using UHPLC-QE-MS to investigate the impact of DSS on the metabolites in the brains of AD rats. The quality control of untargeted metabolomics in brain tissues using UHPLC-QE-MS was well-maintained (Supplementary File S6). The detected metabolites in rat brain tissues were classified into 13 categories, with lipids and lipid metabolism molecules comprising the highest proportion at 28.696% (Figure 4A). PCA (Figure 4B) and OPLS-DA (Figures 4C, D) analyses revealed distinct separation of metabolites among the three groups, indicating significant metabolic disruptions in AD rats, partially restored through DSS treatment.

Utilizing Student’s t-test (*p* < 0.05) and VIP values (VIP > 1, Variable Importance in the Projection of the first principal component in the OPLS-DA model), we identified 338 differential metabolites (171 upregulated and 167 downregulated) between the Control and Model groups (Figure 4E; Supplementary File S7). Additionally, the DSS and Model groups exhibited 540 differential metabolites (231 upregulated and 309 downregulated) (Figure 4F; Supplementary File S7). Quantitative analysis of the differential metabolites, log-transformed with a base of 2, revealed that, compared to the Control, Ophthalmic acid (OA) (increased) and 3-Sulfinoalanine (decreased) were the most significantly altered metabolites in the Model group. In comparison to the Model group, the DSS group showed significant alterations in OA (decreased) and Phosphocreatine (PCr) (increased) (Figures 4G, H). These metabolites fall under the category of Carboxylic acids and derivatives, specifically Organic acids and derivatives. This suggests a notable influence of DSS treatment on the relative abundance of brain tissue metabolites, particularly Organic acids and derivatives, with significant improvement observed in OA following DSS intervention. Cluster analysis further explored the differences in various classes of Organic acids and derivatives (Figure 4I; Supplementary File S8), indicating overall improvements in these metabolites after DSS intervention. These results suggest that DSS may exert its therapeutic effects against AD by ameliorating processes related to Organic acids and derivatives.

### 4.5 DSS ameliorates the interplay between gut microbiota and brain tissue metabolites in AD rats

**FIGURE 5:**
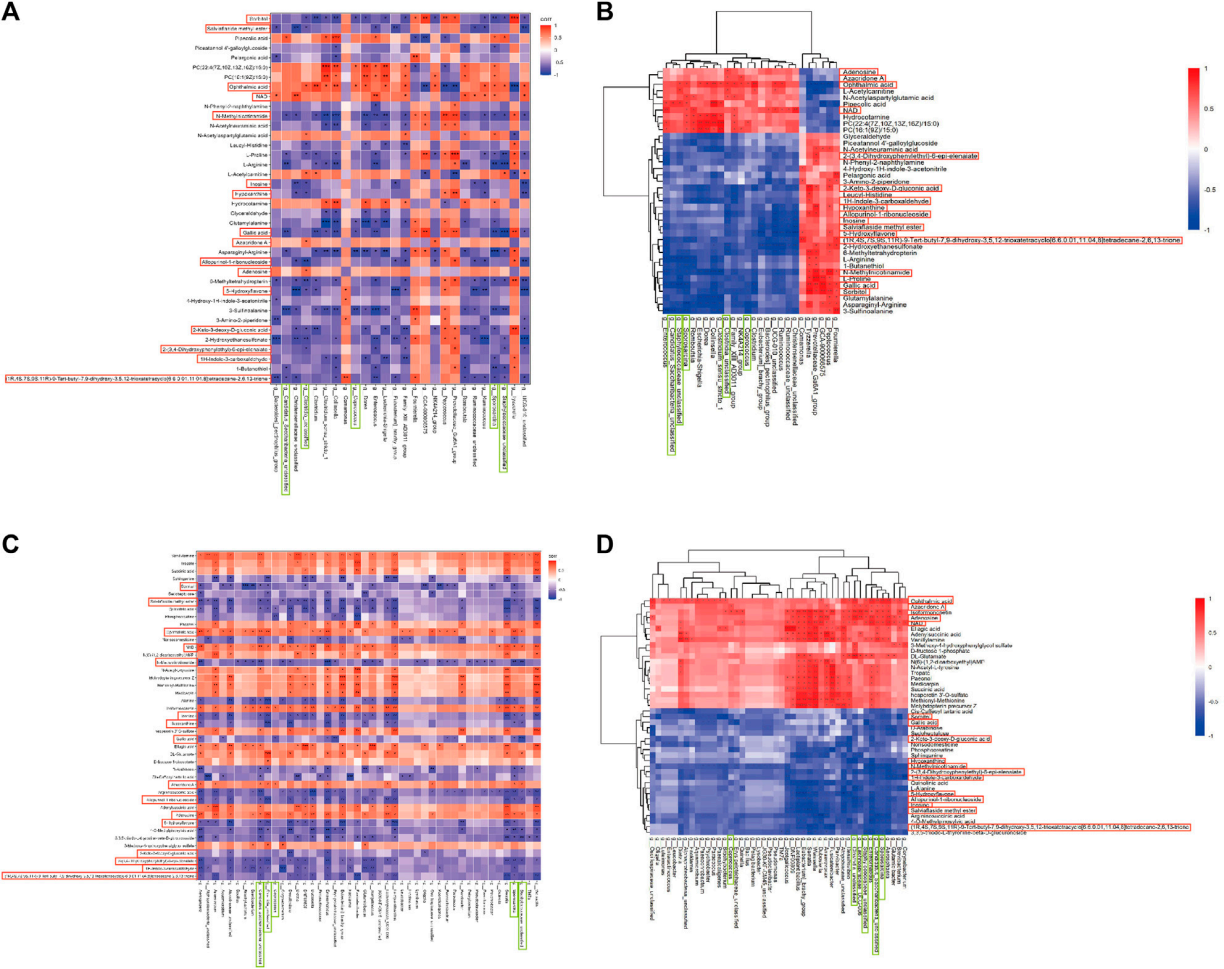
Joint Analysis of Metabolomics and 16S rDNA. **(A)** Spearman Correlation Analysis between Con and M groups. **(B)** Spearman Analysis Heatmap between Con and M groups. **(C)** Spearman Correlation Analysis between M and DSS groups. **(D)** Spearman Analysis Heatmap between M and DSS groups. *p < 0.05; **p < 0.01; ***p < 0.001; ****p < 0.0001.

Spearman correlation analysis was conducted between gut microbiota and brain tissue metabolites to further elucidate the impact of DSS on the interaction between these two factors and its resulting anti-AD effects (Figure 5; Supplementary File S9). Notably, 16 common differential metabolites were identified between the differentially metabolized Con and M groups and the DSS and M groups (highlighted in red boxes in Figure 5), including Adenosine, Allopurinol-1-ribonucleoside, Inosine, and NAD. Additionally, five shared gut microbiota species were observed (highlighted in green boxes in Figure 5), including Sporosarcina, Candidatus_Saccharibacteria_unclassified, Clostridia_unclassified, Staphylococcaceae_unclassified, and Coprococcus.

Further analysis revealed significant correlations (*p* < 0.05) in the Con vs. DSS group comparison, where Candidatus_Saccharibacteria_unclassified exhibited negative correlations with three metabolites and positive correlations with one metabolite; Clostridia_unclassified showed negative correlations with eight metabolites and positive correlations with three metabolites; Coprococcus displayed negative correlations with two metabolites and positive correlations with one metabolite; Sporosarcina had negative correlations with six metabolites and positive correlations with two metabolites; Staphylococcaceae_unclassified showed negative correlations with five metabolites and positive correlations with one metabolite. In the DSS vs. M group comparison, Candidatus_Saccharibacteria_unclassified exhibited negative correlations with nine metabolites and positive correlations with four metabolites; Clostridia_unclassified displayed negative correlations with eight metabolites and positive correlations with four metabolites; Coprococcus showed negative correlations with one metabolite and positive correlations with one metabolite; Sporosarcina exhibited negative correlations with eleven metabolites and positive correlations with four metabolites; Staphylococcaceae_unclassified displayed negative correlations with eight metabolites and positive correlations with four metabolites.

The combined analysis highlighted alterations in the relationships between Candidatus_Saccharibacteria_unclassified and Gallic acid, Azacridone A, Sorbitol, Allopurinol-1-ribonucleoside, Inosine, 5-Hydroxyflavone, 1H-Indole-3-carboxaldehyde, Adenosine, 2-(3,4-Dihydroxyphenylethyl)-6-epi-elenaiate, Salviaflaside methyl ester, NAD, and OA; Clostridia_unclassified and 2-Keto-3-deoxy-D-gluconic acid, 1H-Indole-3-carboxaldehyde, and NAD; Coprococcus and OA, Sorbitol, and Azacridone A; Sporosarcina and 2-Keto-3-deoxy-D-gluconic acid, Hypoxanthine, Azacridone A, Inosine, 5-Hydroxyflavone, Allopurinol-1-ribonucleoside, Salviaflaside methyl ester, Adenosine, and 2-(3,4-Dihydroxyphenylethyl)-6-epi-elenaiate; Staphylococcaceae_unclassified and Sorbitol, Inosine, Salviaflaside methyl ester, Hypoxanthine, Azacridone A, Allopurinol-1-ribonucleoside, Adenosine, and NAD. Among these interactions, Candidatus_Saccharibacteria_unclassified exhibited the most changes, suggesting it may be one of the gut microbiota most influenced by DSS intervention. Azacridone A demonstrated the highest number of altered relationships with various gut microbiota and was statistically significant only in the DSS group, indicating it may be one of the metabolites most influenced by DSS intervention.

### 4.6 DSS modulates metabolic pathways in the brains of AD rats

**FIGURE 6:**
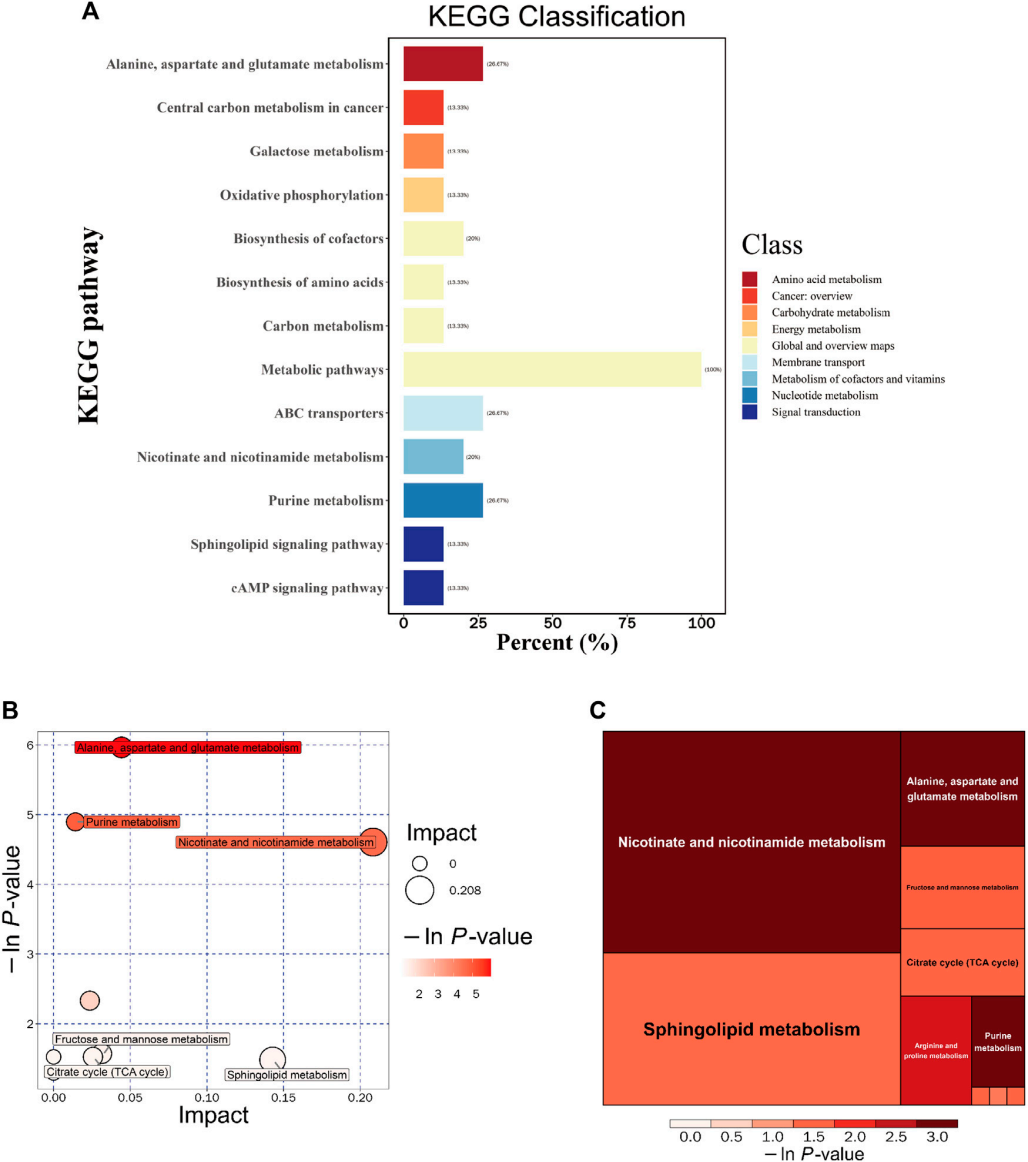
Integrated KEGG Analysis of Metabolic Pathways in Rats. **(A)** KEGG Classification of Differential Metabolites in the DSS and M Groups: The figure displays the KEGG classification of differential metabolites in the DSS group compared to the M group. **(B)** Bubble Chart of Metabolic Pathway Analysis in the DSS Group Compared to the M Group. **(C)** Rectangular Tree Chart of Metabolic Pathway Analysis in the DSS Group Compared to the M Group. *p < 0.05; **p < 0.01; ***p < 0.001; ****p < 0.0001.

To gain further insights into the functional implications and host effects of these differential metabolites, we systematically classified and annotated the identified metabolites using the KEGG database. This allowed us to elucidate their functional characteristics and identify key biochemical and signaling pathways. A comparison of metabolic pathways and the corresponding percentages of differential metabolites between the DSS and M groups is presented in Figure 6A. Notably, the most pronounced differences were observed in the “Metabolic pathways” category, a subset of the “Global and overview maps” pathway, with 100% of metabolites exhibiting disparities.

Through comprehensive analysis of the pathways associated with differential metabolites (including enrichment and topological analyses), we refined our focus to identify key pathways with the highest relevance to metabolite differences (Figures 6B, C; Table 4). Among these, the pathway most strongly correlated with differential metabolites was “Alanine, aspartate, and glutamate metabolism,” featuring differentially expressed metabolites such as Argininosuccinic acid, Adenylsuccinic acid, and Succinic acid. The pathway with the highest number of hits was “Purine metabolism (PM),” encompassing differential metabolites including Inosine, Hypoxanthine, Adenylsuccinic acid, and Adenosine. The pathway with the highest topological impact factor (Impact value) was “Nicotinate and nicotinamide metabolism (NANM),” featuring differential metabolites NAD and Quinolinic acid. These findings provide a comprehensive overview of the impact of DSS on the metabolic pathways in the brains of AD rats.

**TABLE 4:** Metabolic pathway analysis of the DSS group compared to the M group.

Pathway	Total	Hits	Raw p	-ln(p)	Impact	Hits Cpd
Alanine, aspartate and glutamate metabolism	24	3	0.0025634	5.9664	0.0443	Argininosuccinic acid cpd:C03406; Adenylsuccinic acid cpd:C03794; Succinic acid cpd:C00042
Purine metabolism	68	4	0.0074678	4.8972	0.01426	Adenylsuccinic acid cpd:C03794; Adenosine cpd:C00212; Hypoxanthine cpd:C00262; Inosine cpd:C00294
Nicotinate and nicotinamide metabolism	13	2	0.0099838	4.6068	0.20833	Quinolinic acid cpd:C03722; NAD cpd:C00003
Arginine and proline metabolism	44	2	0.097187	2.3311	0.02364	Argininosuccinic acid cpd:C03406; Phosphocreatine cpd:C02305
Fructose and mannose metabolism	19	1	0.20809	1.5698	0.03142	Sorbitol cpd:C00794
Propanoate metabolism	20	1	0.21782	1.5241	0	Succinic acid cpd:C00042
Butanoate metabolism	20	1	0.21782	1.5241	0	Succinic acid cpd:C00042
Citrate cycle (TCA cycle)	20	1	0.21782	1.5241	0.02566	Succinic acid cpd:C00042
Sphingolipid metabolism	21	1	0.22744	1.4809	0.14286	Sphinganine cpd:C00836
Galactose metabolism	26	1	0.2739	1.295	0	Sorbitol cpd:C00794

### 4.7 DSS ameliorates the expression of inflammatory factors and oxidative stress levels in the brain tissues of AD rats

**FIGURE 7:**
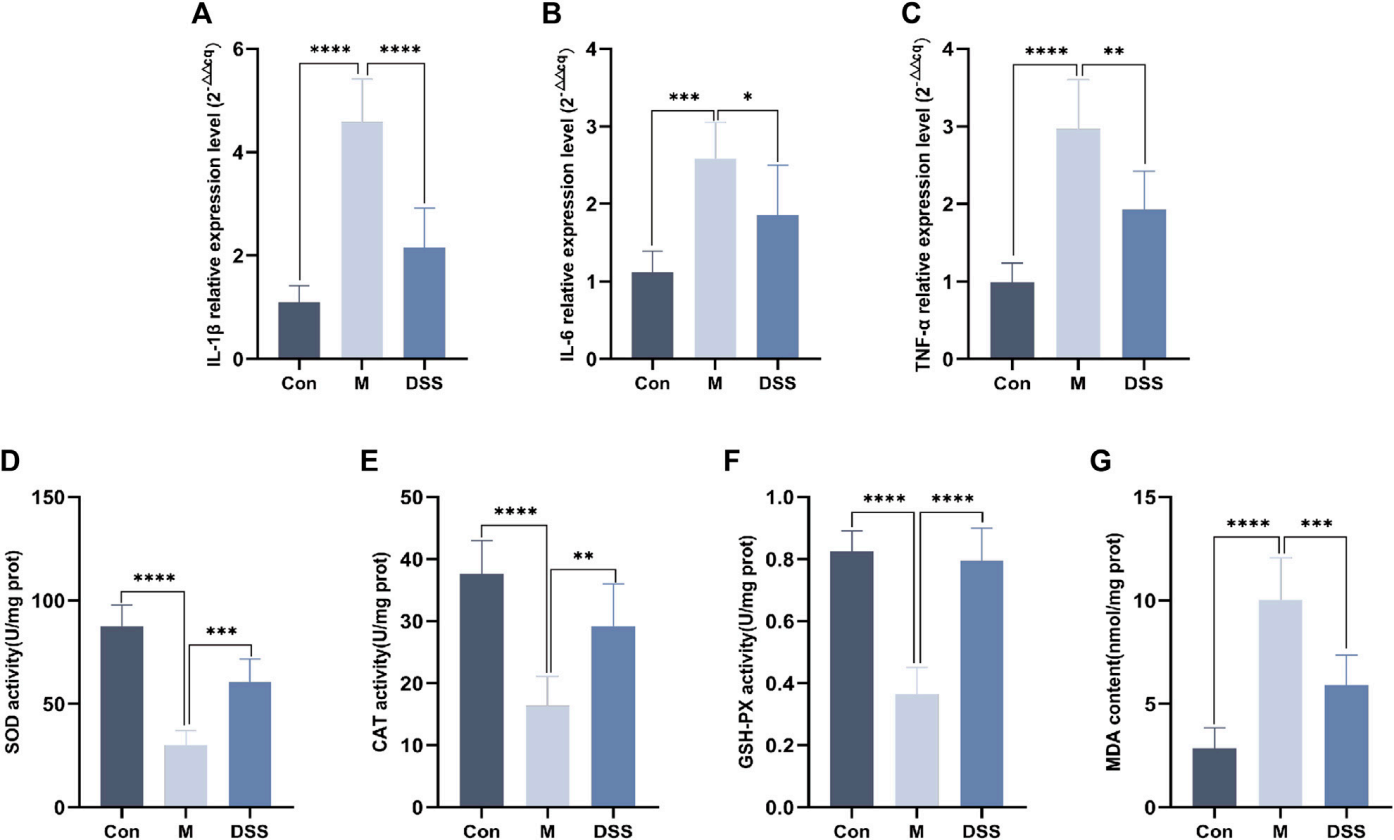
Expression of inflammatory factors and oxidative stress levels in rat brain Tissues. **(A)** IL-1β relative expression Level. **(B)** IL-6 relative expression Level. **(C)** TNF-α relative expression Level. **(D)** SOD Content. **(E)** CAT Content. **(F)** GSH-PX Content. **(G)** MDA content. *p < 0.05; **p < 0.01; ***p < 0.001; ****p < 0.0001.

Building upon the findings from 16S rDNA and metabolomics analyses, the results point towards the efficacy of DSS in reducing neuroinflammation and oxidative stress in AD rats. Consequently, we utilized RT-qPCR to assess the levels of inflammatory factors and measured the inhibition rate and activity of SOD. The RT-qPCR results revealed (Figures 7A–C) that, compared to the Control group, the expression of inflammatory factors IL-1β, IL-6, and TNF-α increased in the Model group, whereas DSS intervention led to a significant reduction in the expression of IL-1β, IL-6, and TNF-α (*p* < 0.05). The evaluation of SOD, CAT, MDA, and GSH-PX levels (Figures 7D–G) showed that, compared to the Control group, the Model group exhibited decreased levels of SOD, CAT, and GSH-PX, along with an increase in MDA levels. Following DSS intervention, there was a significant elevation in SOD, CAT, and GSH-PX levels (*p* < 0.05), accompanied by a decrease in MDA levels, indicating that DSS reversed the oxidative stress levels in AD rats.

## 5 Discussion

In the face of a globally aging population, AD emerges as a significant age-related dementia challenge (Breijyeh and Karaman, 2020). Research increasingly suggests that the microbiota-gut-brain axis (MGBA) plays a crucial role, intertwining metabolism, endocrinology, and immunity (Martins, 2019), and linking gut microbiota with the central nervous system (Yuan et al., 2022). DSS, a revered traditional Chinese medicine, displays neuroprotective and metabolic regulatory attributes. This positions DSS as a promising candidate for counteracting AD through its modulation of the MGBA, offering a potential therapeutic pathway.

In this study, we initiated an investigation into the bloodstream components of DSS. The results revealed that Benzene and substituted derivatives were the predominant constituents. Existing research has underscored the broad-spectrum antimicrobial properties within Benzene and substituted derivatives, specifically in optimizing the structure of (salicylideneamino) benzoic acids (Krátký et al., 2021). These compounds have been recognized as potential inhibitors of Gram-positive bacteria, a classification to which Dubosiella belongs. This elucidates the lower prevalence of Dubosiella in the DSS group and provides an explanation for the observed decrease in gut microbial abundance within this group. The most abundant subclass of compounds identified was Amino acids, peptides, and analogues. Within peptides, mitochondrial-derived peptide (MDP) MOTS-c was found to play a role in aging. Among the eight identified MDPs, MOTS-c, transcribed from the SORF sequence found in mtRNA genes encoding 12s rRNA and 16s rRNA (Lee et al., 2015), comprises 51 base pairs and translates into a 16-amino acid peptide sequence (MRWQEMGYIFYPRKLR) known as MOTS-c (Harhay et al., 2005). Damaged mitochondria generate reactive oxygen species, leading to oxidative stress, cell death, and cognitive impairment in AD (Loera-Valencia et al., 2019). Studies indicate that MOTS-c, through AMP-activated protein kinase (AMPK) phosphorylation, reduces the activation of astrocytes and microglia while diminishing the production of pro-inflammatory cytokines. This mechanism enhances the formation of object and location recognition memory (Jiang et al., 2021). This interpretation aligns with the behavioral outcomes observed in our study through the Morris water maze experiment, where DSS intervention significantly improved learning and spatial memory abilities in AD rats. Concurrently, histological assessments using HE staining and Nissl staining revealed that DSS restored the morphology of hippocampal neurons in AD rats, providing evidence of the neuroprotective effects of DSS on the brain tissue of AD rats.

In our study, 16S rDNA sequencing revealed that DSS significantly reduced intestinal microbial community abundance in AD rats. Notably, the microbial species varied distinctly across the three groups, highlighting DSS’s impact on gut microbiota composition. Ligilactobacillus, prevalent in the DSS group and scarce in the model group, emerged as a key probiotic species. Ligilactobacillus, identified as a motile lactic acid bacterium within the animal gastrointestinal tract (Suzuki et al., 2023), belongs to the category of probiotics. Research underscores probiotics’ role in immunomodulation, stress resistance, pathogen defense, and intestinal barrier enhancement (Suez et al., 2019). Probiotic interventions in mice have shown improved spatial memory, reduced hippocampal Aβ plaques (Abraham et al., 2019; Ma et al., 2023), and enhanced synaptic plasticity (Rezaei AslZahra and Salami, 2019). A clinical trial further substantiates these findings, indicating improved cognitive function and favorable alterations in plasma biomarkers in AD patients subjected to probiotic supplementation (Leblhuber et al., 2018), suggesting DSS’s potential anti-AD effects through beneficial gut microbial modulation.

In our metabolite investigation, DSS exhibited a marked regulatory effect on organic acids and derivatives, particularly in reducing OA and elevating PCr levels. Elevated OA levels, a biomarker linked to aging, oxidative stress, and cognitive decline (Chaleckis et al., 2016; Kameda et al., 2020), were notably diminished by DSS. PCr, essential for providing energy to muscles and nerve cells, emerged as a focal point of DSS’s regulatory effects. Previous research suggests that increased PCr levels can mitigate plasma neuroinflammatory marker GFAP and decrease Aβ uptake, thereby lowering the risk of AD (Tian et al., 2023). Furthermore, PCr, through modulation of the AKT/GSK-3β/CDK5 pathway, exhibits neuroprotective properties by alleviating Aβ protein toxicity and shielding nerve cells from damage (Ai et al., 2021). Metabolomic analysis in our study further corroborates these findings, demonstrating a reduction in OA and an elevation in PCr levels in brain tissues following DSS intervention, thus highlighting the anti-AD efficacy of DSS.

In the combined analysis of gut microbiota and brain tissue metabolites, Candidatus Saccharibacteria emerged as the microbial group exhibiting the most significant variations associated with metabolites (Zhang et al., 2023). Its role may be intricately linked to inflammation, evident in its significant positive correlation with OA. The pro-inflammatory effects of Candidatus Saccharibacteria are speculated to contribute to the regulation of the aging process and cognitive impairments through the MGBA. Azacridone A, identified as a metabolite undergoing the most significant changes across various gut microbiota, demonstrated statistically significant differences exclusively in the DSS group. With potential pharmacological activity, Azacridone A is likely associated with oxidative stress and pro-inflammatory responses (Scopton and Kelly, 2004). Our study revealed a positive correlation between Azacridone A and four distinct microbial groups, suggesting that DSS potentially modulates gut microbiota and, through the MGBA, regulates metabolic responses such as inflammation and oxidative stress in brain tissues, contributing to its anti-AD effects.

This inference was substantiated through a comprehensive analysis employing the Kyoto Encyclopedia of Genes and Genomes (KEGG) database. The PM, identified as the pathway with the highest count of differentially expressed metabolites, has been consistently reported in previous studies examining brain tissue samples from both APP/PS1 transgenic mice and deceased AD patients (González-Domínguez et al., 2015; Alonso-Andrés et al., 2018; Mahajan et al., 2020). In particular, metabolites associated with PM, such as Inosine, play a crucial role in AD. Inosine, typically derived from the breakdown of Adenosine (Teixeira et al., 2020), serves as an intermediate product in the PM and exhibits increased levels across various AD animal models (González-Domínguez et al., 2015; Alonso-Andrés et al., 2018). Conversely, researchers have observed that Inosine can ameliorate aging and cognitive impairments (Teixeira et al., 2020; Ruhal and Dhingra, 2018). In the STZ-induced AD rat model used in our study, Inosine demonstrated improvements in memory, reduced acetylcholinesterase activity, and displayed antioxidative properties (Teixeira et al., 2020). This biphasic outcome underscores potential issues related to the efficacy of Inosine. Our study proposes that DSS diminishes the utilization of Inosine, leading to its accumulation and consequently exerting antioxidative effects, thereby enhancing cognitive function in AD rats. Notably, the NANM, identified as the pathway with the highest topological impact in the network analysis, is closely linked to nicotinamide adenine dinucleotide (NAD+) and its association with AD. NAD+ is a vital coenzyme involved in various metabolic reactions, including glycolysis, the tricarboxylic acid (TCA) cycle, and oxidative phosphorylation (Xing et al., 2019). The depletion of NAD+ is correlated with the aging process, and its exhaustion is implicated in the pathogenesis of AD (Lautrup et al., 2019). Dysregulation of NANM has been observed in AD mouse models such as 5×FAD (Kim et al., 2020), APP/PS1 (Trushina et al., 2012), and 3×TgAD (Zhao et al., 2021), as well as in fibroblasts from late-onset AD patients, highlighting its relevance to AD (Sonntag et al., 2017), NAD + and its precursor substances improve on cognitive function in AD mouse models (Xie et al., 2019). Our research indicates that the improvement in cognitive function in AD rats following DSS intervention may be mediated through the NAD+ pathway via NANM.

The results from both 16S rDNA sequencing and metabolomics consistently point towards the anti-inflammatory and antioxidant properties of DSS, Correlating with Neuroinflammation and Oxidative Stress in AD Brain (PENG et al., 2022). To further substantiate these findings, we conducted RT-qPCR to assess the levels of inflammatory factors and measured the contents of key antioxidants, including SOD, CAT, GSH-PX, and MDA. Our findings collectively reinforce the notion that DSS effectively suppresses inflammation and oxidative stress levels in AD rats.

While our study has elucidated the therapeutic potential of DSS in AD, it is important to acknowledge its limitations. The complexity of DSS as a TCM formulation poses challenges in isolating and attributing specific effects to individual constituents (Song et al., 2020; Luo et al., 2023). Additionally, our use of the STZ-induced AD rat model, while beneficial, differs from genetically modified models typically used in AD research, potentially affecting the generalizability of our findings (Dhapola et al., 2023; Kadhim et al., 2022). Future research could benefit from employing diverse AD models, including transgenic mice, to broaden the understanding of AD pathologies (Sharma and Martins, 2023). Advanced analytical techniques like metabolomics and pharmacokinetics are suggested to deepen our knowledge of DSS’s bioavailability and pharmacological actions.

## 6 Conclusion

This study employs a comprehensive approach, encompassing behavioral analysis, histopathology, 16S rDNA sequencing, and metabolomics, to elucidate DSS’s efficacy against AD.

The findings reveal DSS as a multifaceted therapeutic agent, effectively reducing cognitive impairment and AD-related brain pathology. Noteworthy among our discoveries is DSS’s remarkable ability to rectify dysbiosis within the gut microbiota, offering a promising avenue for modulating the MGBA. Specifically, DSS exerts significant regulatory effects on the Ligilactobacillus population, pivotal in maintaining gut health and known for its probiotic attributes. Additionally, our exploration of metabolic disruptions in AD rats reveals that DSS orchestrates processes related to PM and NANM metabolism, unveiling potential therapeutic targets. Of particular significance is the observed interplay between Candidatus Saccharibacteria and OA, shedding light on DSS’s ability to modulate inflammation and oxidative stress through the MGBA. This work highlights DSS’s potential as an innovative therapeutic approach, addressing cognitive decline, neuropathological changes, and gut dysbiosis in AD, and underscores the need for further research into its molecular mechanisms.

## Data Availability

The datasets presented in this study can be found in online repositories. The names of the repository/repositories and accession number(s) can be found below: https://www.ncbi.nlm.nih.gov/, PRJNA1029816.

## Abbreviations

DSS, Danggui Shaoyao San; AD, Alzheimer’s disease; STZ, streptozotocin; MWM, Morris Water Maze; SOD, superoxide dismutase; CAT, catalase; GSH-PX, glutathione peroxidase; MDA, malondialdehyde; OA, Ophthalmic acid; PCr, Phosphocreatine; PM, Purine metabolism; TCM, Traditional Chinese medicine; MGBA, Microbial-Gut-Brain Axis; NANM, Nicotinate and nicotinamide metabolism.
