# Supplementary material for: Danggui Shaoyao San: comprehensive modulation of the microbiota-gut-brain axis for attenuating Alzheimer’s disease-related pathology
Source: Front Pharmacol. 2024 Jan 12;14:1338804. doi: 10.3389/fphar.2023.1338804 (PMC10811133; doi:10.3389/fphar.2023.1338804)
Supplement: Supplementary file 5 [file DataSheet1.PDF]

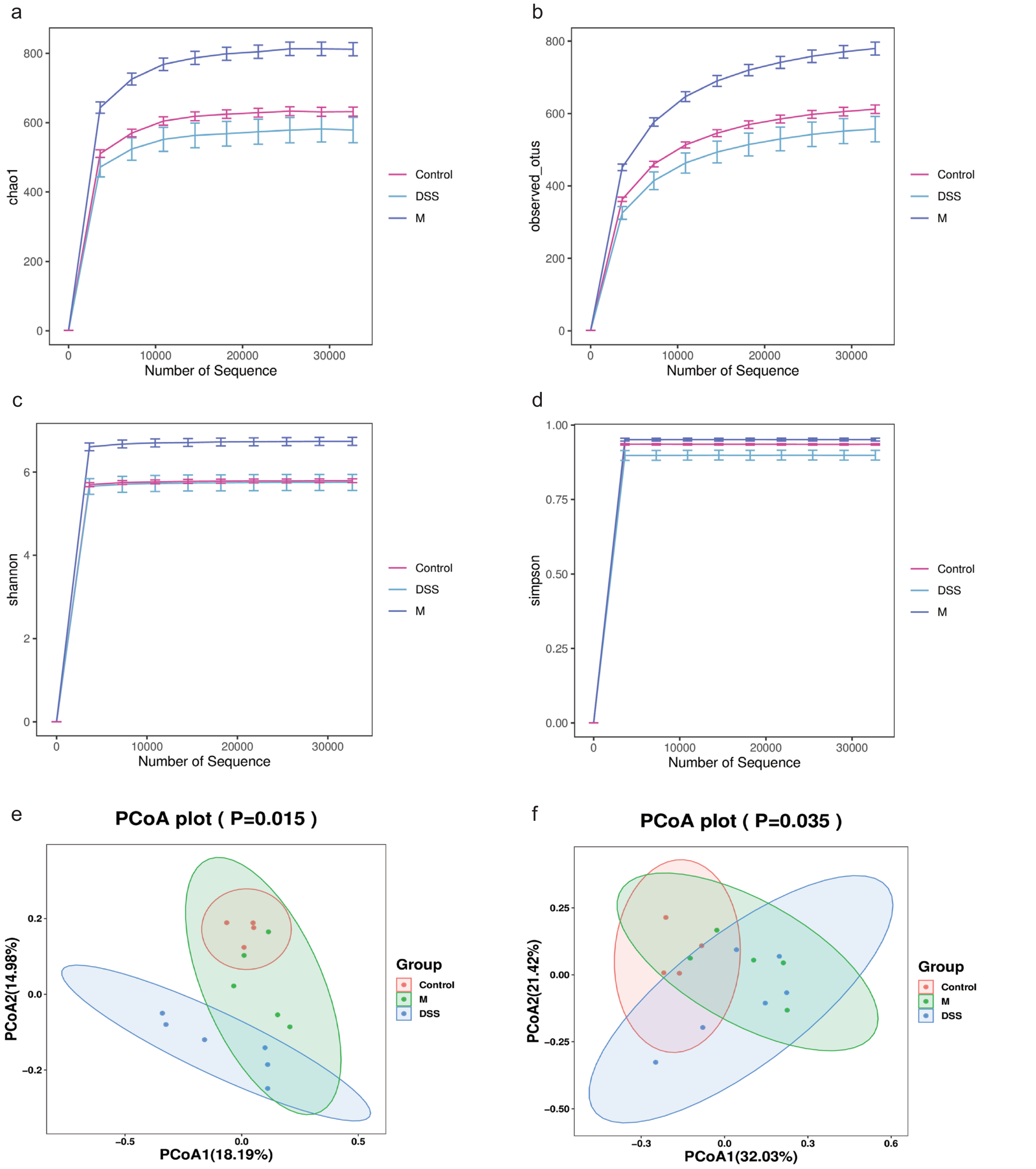

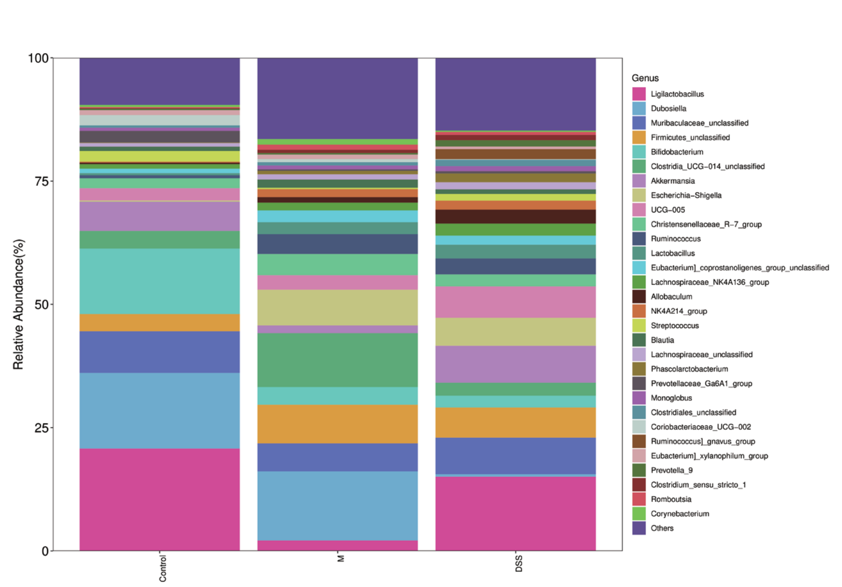

a. Chao1 Index

b. observed species Index

c.Shannon Index

d.Simpsond Index

e.PcoA-unweighted unifrac

f.PcoA-weighted unifrac

g.Genus-level community distribution percentage
