# Supplementary material for: Danggui Shaoyao San: comprehensive modulation of the microbiota-gut-brain axis for attenuating Alzheimer’s disease-related pathology
Source: Front Pharmacol. 2024 Jan 12;14:1338804. doi: 10.3389/fphar.2023.1338804 (PMC10811133; doi:10.3389/fphar.2023.1338804)
Supplement: Supplementary file 9 [file Table6.DOCX]

**Quality Control in Untargeted Metabolomics Analysis Using UHPLC-QE-MS**

Untargeted metabolomics analysis of rat cortical samples was performed using UHPLC-QE-MS. Three visualization methods, namely Total Ion Current (TIC), Extracted Ion Chromatogram (EIC), Principal Component Analysis (PCA), and QC sample correlation analysis, were employed to ensure data quality. High-resolution mass spectrometry was utilized to collect data from both positive and negative ion modes for improved metabolite coverage.

In both positive and negative ion modes (Fig. 1-a,b), the TIC baselines were clear, and the peaks were well-defined in terms of number, position, and intensity. The response peak height differences of internal standards among QC samples were examined to assess the stability of the detection (Fig. 1-c,d), demonstrating good retention time and response intensity stability of internal standards in QC samples. This indicates the stability of the instrument data acquisition.

The detection of substance residues during the entire experiment was examined by analyzing blank samples interspersed throughout the experiment. Figure 1-e,f shows that no significant peaks were detected in any of the blank samples for all internal standards, indicating effective control of substance residues, and cross-contamination between samples was within a manageable range.

The PCA score plot illustrates good clustering of QC samples (Fig. 2-a), with all samples clustering near the origin, indicating good stability. Figure 2-b demonstrates that all QC samples fall within ±2 STD, indicating high-quality data for this experiment. The closer the correlation of QC samples is to 1 (Fig. 2-c), the better the stability of the entire method and the higher the data quality. In this experiment, QC sample correlation was >0.9, indicating high data quality (Fig. 2-c).


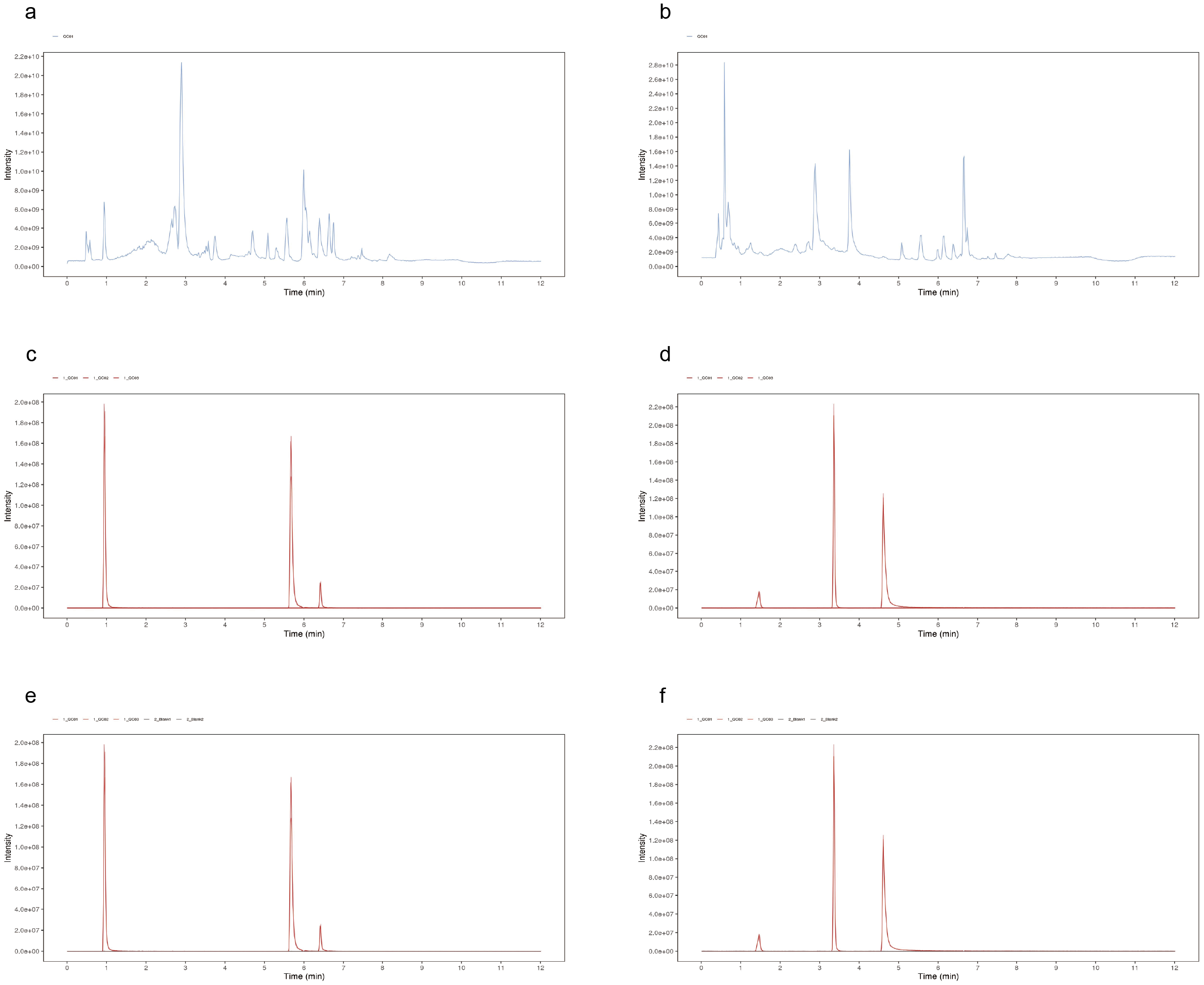


**Figure 1** Quality Control in UHPLC-QE-MS Untargeted Metabolomics Analysis. (a. TIC in Positive Ion Mode for QC Samples in UHPLC-QE-MS Analysis.b. TIC in Negative Ion Mode for QC Samples in UHPLC-QE-MS Analysis.c. EIC of Internal Standards in Positive Ion Mode for All QC Samples.d. EIC of Internal Standards in Negative Ion Mode for All QC Samples.e. EIC of Internal Standards in Positive Ion Mode for Blank and QC Samples.f. EIC of Internal Standards in Negative Ion Mode for Blank and QC Samples.)


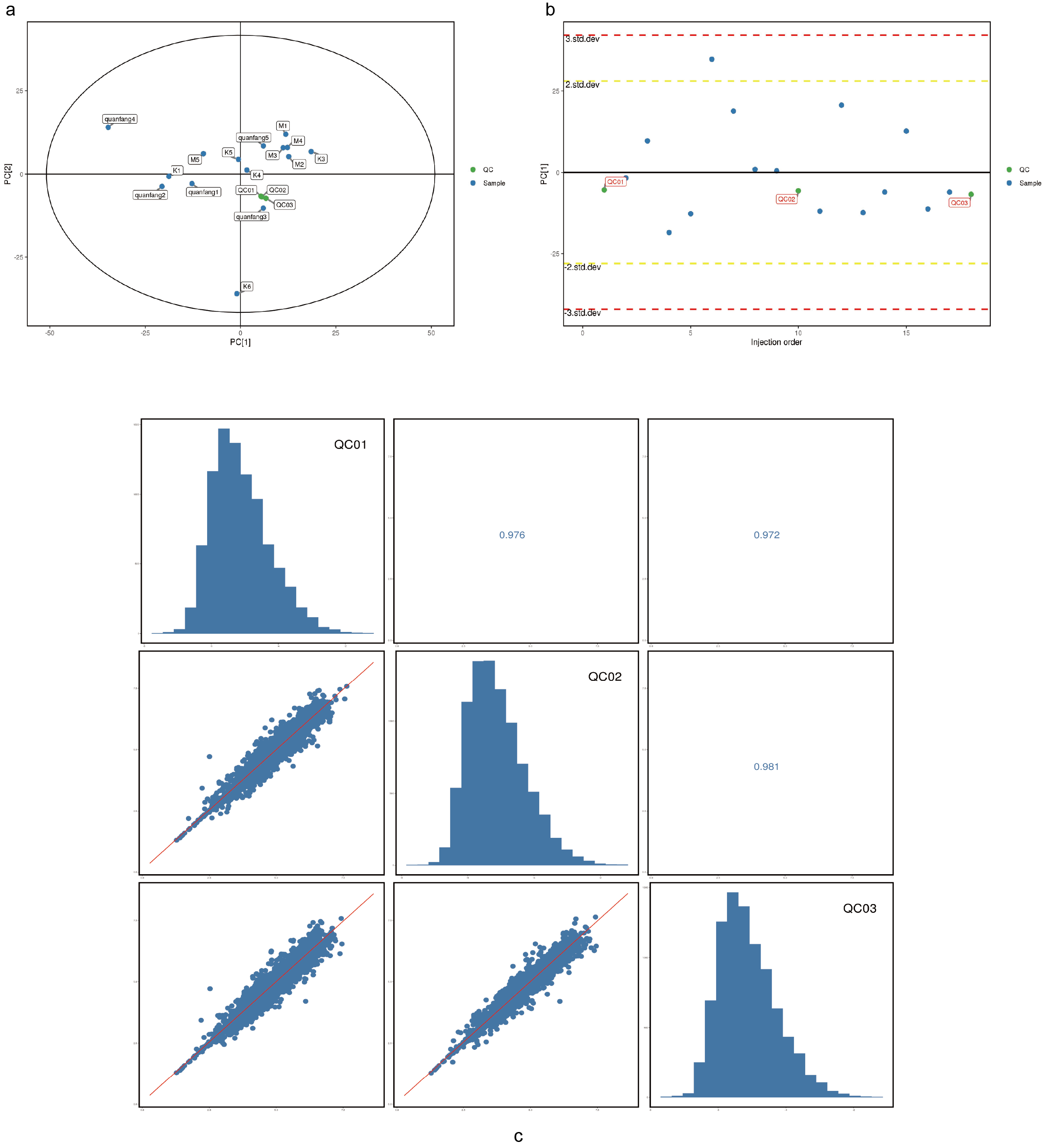


**Figure 2** Quality Control in UHPLC-QE-MS Untargeted Metabolomics Analysis. (a. PCA Score Plot.b. PCA-X Univariate Distribution Plot for QC Samples.c. Correlation Analysis of QC Samples.)
